# Data on sea surface biophysical parameters during different monsoon seasons

**DOI:** 10.1016/j.dib.2019.104982

**Published:** 2019-12-16

**Authors:** Md Suffian Idris, Hing Lee Siang, Roswati Md Amin

**Affiliations:** School of Science and Marine Environment, Universiti Malaysia Terengganu, 21030 Kuala Nerus, Terengganu, Malaysia

**Keywords:** Ocean biophysical data, Monsoon season, Peninsular Malaysia, South China sea

## Abstract

The biophysical data presented in this article were collected in the east coast of Peninsular Malaysia from May to November 2009. These monthly surface data were obtained from 32 stations along the coastal-offshore transect and were analyzed to understand the spatial and temporal distributions of biophysical parameters during different monsoon seasons. The data presented here include sea surface temperature (SST), sea surface salinity (SSS), Secchi disk depth (SDD), Chlorophyll-a (Chl-a), suspended particulate matter (SPM), mineral suspended solid (MSS) and chromophoric dissolved organic matter (CDOM).

**Table:** Specifications Table

Subject	Earth and Planetary Sciences
Specific subject area	Oceanography
Type of data	Tables Figures
How data were acquired	YSI 6600 Multi Parameter V2 Sonde and SBE 911 CTD profiler for surface salinity and temperature; Cary-100 (Cary Instruments) double beam spectrophotometer for Chlorophyll-a and chromophoric dissolved organic matter (CDOM); Secchi disk for Secchi disk depth (SDD)
Data format	Raw Analyzed
Parameters for data collection	Sea surface salinity (SSS); Sea surface temperature (SST); Chlorophyll-a (Chl-a), Suspended particulate matter (SPM); Mineral suspended solid (MSS); chromophoric dissolved organic matter (CDOM); Secchi disk depth (SDD)
Description of data collection	For Chl-a: Water samples were filtered onsite using 0.7 μm GF/F filters and store frozen. For SPM and MSS: Water samples were filtered onsite using 0.7 μm GF/F filters, store frozen and dried weight were obtained. For CDOM: Water samples were filtered onsite using 0.2 μm Nucleopore polycarbonate filters and store frozen.
Data source location	Data were collected at 32 stations in the east coast of Peninsular Malaysia (5° 02–58′ N to 102° 45’ – 103° 35′ E) from May to November 2009
Data accessibility	Data are provided within this article

**Table:** 

**Value of the Data** • The data allow the investigation of spatial and temporal variations in biophysical parameters. • The data can be used to study the changes in biophysical parameters during different monsoon seasons. • The data can be used to compare and validate satellite ocean colour measurements in this data-scarce tropical region. • The dataset can be used in bio-optical models pertaining to the conservative mixing behaviour of CDOM with salinity.

## 1 Data

**Fig. 1:**
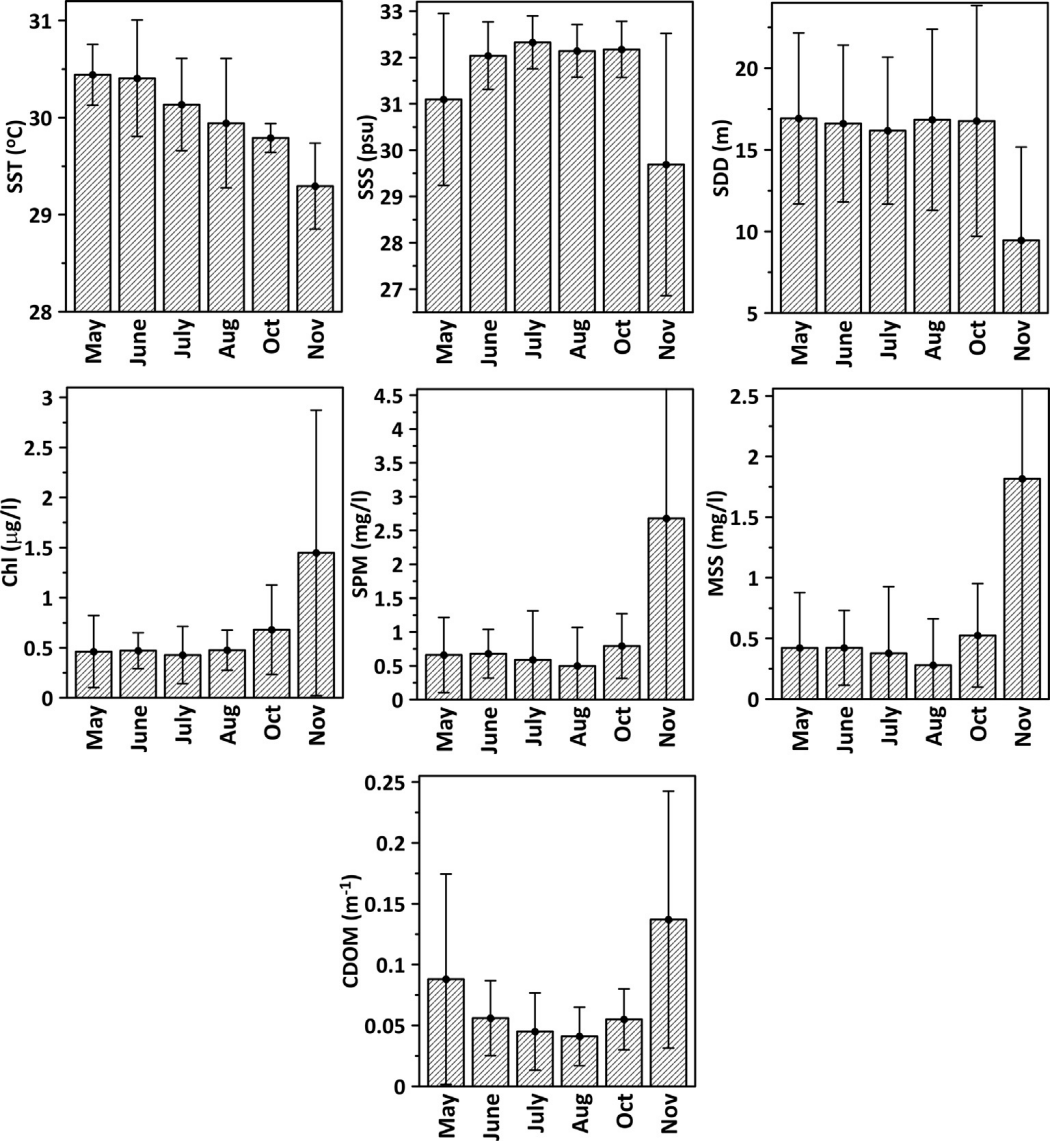
Monthly mean values of surface biophysical parameters (SST, SSS, SDD, Chl, SPM, MSS and CDOM) in the east coast of Peninsular Malaysia during May–Nov 2009.

**Fig. 2:**
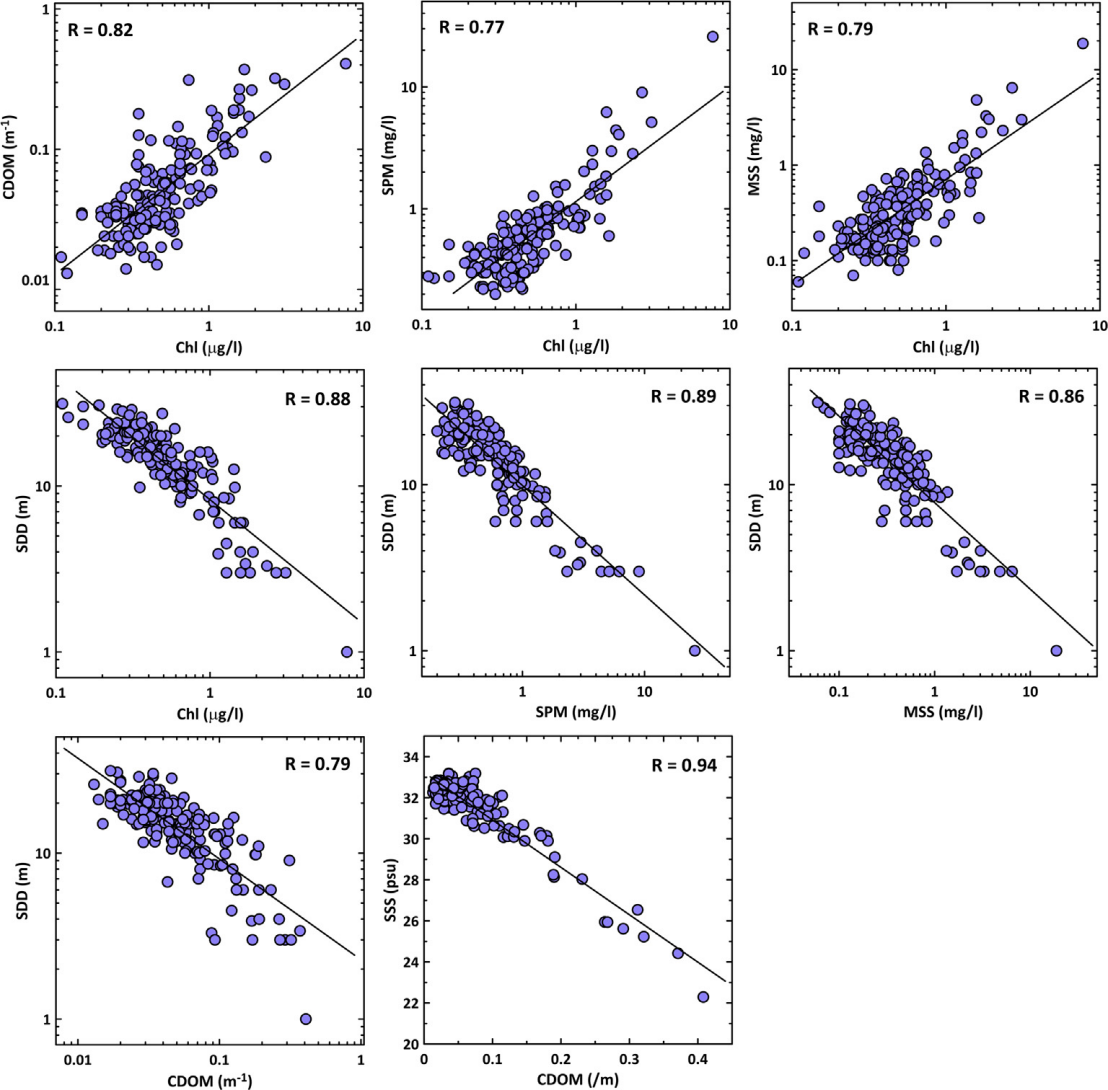
The interrelationships among certain biophysical parameters.

The dataset included in this article consists of 2 Tables and 1 Figure that represent the spatio-temporal distribution of surface biophysical parameters. Table 1 summarizes the details of the sampling locations (longitude and latitude) and depths. The monthly variations sea surface temperature (SST), sea surface salinity (SSS), Secchi disk depth (SDD), chlorophyll-a (Chl-a), suspended particulate matter (SPM), mineral suspended solid (MSS) and chromophoric dissolved organic matter (CDOM) are presented in Table 2 and Fig. 2. SST and SSS during the measurement period ranged from 28.2 °C to 32.0 °C and from 22.3 psμ to 33.2 psμ, respectively. Relatively high SST and SSS were recorded in May and July, respectively, while low values for both parameters were measured in November. Similarly, relatively low SDD was recorded in November and there were considerable fluctuations between the monsoon seasons, ranging from 1.0 m to 31.3 m (October) (Table 2 and Fig. 1). All optically active constituents (Chl-a, SPM, MSS and CDOM) showed clear seasonal variations, with minimum values during the inter-monsoon (May and October) and Southwest monsoon (June to August) and maximum values during the northeast monsoon (November). Chlorophyll-a concentrations varied between 0.11 μg/l (October) and 7.74 μg/l (November) and SPM between 0.20 (August) and 22.80 mg/l (November). The MSS ranged between 0.06 mg/l (October) and 18.80 mg/l (November) whereas CDOM varied between 0.01 m^−1^ (May) and 0.41 m^−1^ (Table 2 and Fig. 1).

**Table 1:** Location of sampling stations.

Station	Longitude (E)	Latitude (N)	Depth (m)
St1	102° 45′ 34.3″	5° 41′ 11.4″	14.0
St2	102° 47′ 19.8″	5° 43′ 13.3″	21.4
St3	102° 49′ 02.7″	5° 45′ 12.5″	23.0
St4	102° 52′ 03.1″	5° 48′ 40.1″	31.5
St5	102° 54′ 52.5″	5° 51′ 54.1″	41.2
St6	102° 57′ 40.6″	5° 55′ 09.2″	46.2
St7	103° 00′ 29.6″	5° 58′ 22.3″	43.5
St14	103° 00′ 39.1″	5° 31′ 37.1″	17.7
St15	103° 02′ 26.4″	5° 33′ 36.9″	19.6
St16	103° 04′ 05.8″	5° 35′ 32.7″	18.3
St17	103° 07′ 10.4″	5° 39′ 02.0″	36.8
St18	103° 09′ 59.9″	5° 42′ 14.6″	49.0
St19	103° 12′ 50.2″	5° 45′ 29.9″	53.7
St20	103° 15′ 40.0″	5° 48′ 42.7″	51.0
St28	103° 12′ 09.9″	5° 18′ 36.5″	16.9
St29	103° 13′ 54.0″	5° 20′ 34.5″	27.5
St30	103° 15′ 37.9″	5° 22′ 35.7″	40.3
St31	103° 18′ 40.2″	5° 26′ 02.5″	47.9
St32	103° 21′ 30.9″	5° 29′ 15.9″	52.7
St33	103° 24′ 18.0″	5° 32′ 27.5″	55.0
St34	103° 27′ 08.5″	5° 35′ 41.8″	55.0
St42	103° 20′ 19.1″	5° 02′ 25.4″	21.0
St43	103° 22′ 02.7″	5° 04′ 23.4″	32.0
St44	103° 23′ 45.6″	5° 06′ 19.9″	38.0
St45	103° 26′ 41.6″	5° 09′ 39.9″	48.0
St46	103° 29′ 37.7″	5° 13′ 00.2″	52.0
St47	103° 32′ 25.4″	5° 16′ 11.5″	56.7
St48	103° 35′ 21.5″	5° 19′ 31.4″	57.3
St49	103° 12′ 28.4″	5° 23′ 46.8″	26.5
St50	103° 11′ 35.2″	5° 22′ 45.9″	20.8
St51	103° 10′ 37.9″	5° 21′ 46.6″	17.1
St52	103° 09′ 45.0″	5° 20′ 48.6″	11.2

**Table 2:** Surface biophysical data collected during May–August and October–November 2009.

Month	Station	SST (^o^C)	SSS (psu)	SDD (m)	Chl-a (μg/l)	SPM (mg/l)	MSS (mg/l)	CDOM (m^−1^)
May	St14	30.68	30.15	10.0	0.350	0.690	0.490	0.179
	St15	31.13	32.05	10.0	0.610	0.620	0.390	0.110
	St16	30.52	32.10	12.0	0.440	0.790	0.420	0.062
	St17	31.30	31.87	16.0	0.380	0.490	0.130	0.040
	St18	30.01	31.82	21.5	0.330	0.420	0.260	0.056
	St19	30.25	31.92	22.0	0.240	0.480	0.250	0.034
	St20	30.54	32.21	21.0	0.310	0.320	0.160	0.019
	St 28	30.48	26.54	9.0	0.740	1.530	1.360	0.312
	St 29	30.61	30.08	13.5	0.420	0.680	0.480	0.116
	St 30	30.42	30.17	16.5	0.350	0.520	0.420	0.126
	St31	30.14	31.52	18.5	0.200	0.490	0.230	0.036
	St32	30.47	31.94	20.0	0.300	0.410	0.110	0.037
	St33	30.99	32.01	23.5	0.150	0.510	0.370	0.035
	St34	30.76	32.26	26.0	0.120	0.270	0.120	0.013
	St42	30.21	31.91	16.5	0.350	0.520	0.290	0.091
	St43	30.32	31.77	19.5	0.270	0.320	0.170	0.023
	St44	30.31	31.73	21.0	0.250	0.300	0.150	0.033
	St45	30.23	31.85	21.5	0.310	0.500	0.360	0.033
	St46	30.09	31.93	20.0	0.290	0.470	0.220	0.053
	St47	30.12	31.92	21.0	0.300	0.420	0.190	0.029
	St48	30.29	32.15	20.5	0.400	0.420	0.260	0.047
	St49	30.29	31.81	15.0	0.740	0.500	0.300	0.041
	St50	30.36	31.31	15.0	0.550	0.640	0.360	0.115
	St51	30.41	29.90	10.0	1.450	1.210	0.840	0.181
	St52	30.12	24.42	3.5	1.700	2.970	2.210	0.371
June	St1	31.12	30.63	11.5	0.750	0.920	0.390	0.110
	St2	30.24	31.94	15.0	0.410	0.890	0.720	0.067
	St3	30.53	32.33	18.5	0.430	0.460	0.240	0.046
	St4	30.81	32.29	21.5	0.250	0.340	0.180	0.041
	St5	30.89	32.67	20.5	0.200	0.370	0.110	0.033
	St6	31.40	32.35	24.0	0.280	0.360	0.120	0.038
	St7	31.97	31.94	24.0	0.220	0.430	0.150	0.030
	St14	31.24	30.68	12.0	0.630	1.020	0.520	0.145
	St15	30.49	32.54	13.5	0.520	0.660	0.500	0.058
	St16	29.92	32.15	16.5	0.440	0.630	0.530	0.058
	St17	30.39	32.54	18.0	0.370	0.520	0.270	0.041
	St18	30.26	32.54	20.0	0.340	0.440	0.120	0.025
	St19	30.27	32.57	20.5	0.330	0.430	0.170	0.029
	St20	30.27	32.57	29.0	0.310	0.290	0.190	0.027
	St28	30.88	32.59	15.0	0.510	0.850	0.820	0.050
	St29	30.83	31.75	15.5	0.500	0.850	0.450	0.067
	St30	30.69	31.98	11.5	0.530	1.270	0.770	0.056
	St30	30.69	31.98	11.5	0.530	1.270	0.770	0.056
	St31	30.43	32.20	13.5	0.420	0.630	0.390	0.036
	St32	30.05	32.26	17.0	0.380	0.530	0.440	0.034
	St33	30.23	31.90	15.5	0.360	0.470	0.260	0.044
	St34	30.20	32.28	20.0	0.360	0.330	0.230	0.038
	St42	30.50	31.21	13.0	0.700	0.830	0.700	0.092
	St43	30.40	32.54	17.0	0.470	0.630	0.260	0.059
	St44	30.39	32.42	18.0	0.430	0.570	0.470	0.043
	St45	29.99	32.58	17.5	0.510	0.600	0.300	0.033
	St46	28.97	32.34	18.0	0.520	0.520	0.510	0.050
	St47	29.16	33.02	18.5	0.430	0.460	0.100	0.066
	St48	28.97	33.06	22.0	0.400	0.480	0.190	0.041
	St49	30.38	31.54	10.5	0.570	0.790	0.290	0.046
	St50	30.48	30.89	11.5	0.610	0.800	0.600	0.062
	St51	30.62	30.62	9.0	0.760	1.370	1.030	0.073
	St52	30.02	30.29	4.0	1.130	2.030	1.520	0.169
July	St1	30.43	31.77	8.0	0.640	0.870	0.490	0.073
	St2	30.19	31.74	13.0	0.660	0.700	0.520	0.101
	St3	30.59	32.13	17.0	0.330	0.730	0.550	0.033
	St4	30.62	32.12	18.0	0.250	0.320	0.130	0.039
	St5	30.50	32.85	19.0	0.210	0.340	0.150	0.019
	St6	30.61	32.01	22.0	0.220	0.340	0.200	0.038
	St7	30.66	32.01	20.0	0.230	0.330	0.200	0.018
	St14	30.57	31.56	10.0	0.650	0.900	0.670	0.067
	St15	30.60	31.85	12.0	0.530	0.330	0.170	0.058
	St16	30.50	32.25	14.0	0.560	0.370	0.250	0.035
	St17	30.58	32.38	15.5	0.290	0.370	0.230	0.032
	St18	30.53	32.41	18.5	0.280	0.330	0.170	0.035
	St19	30.12	32.37	19.5	0.260	0.300	0.130	0.020
	St20	29.98	32.45	22.5	0.240	0.230	0.170	0.024
	St28	30.55	32.11	12.0	0.670	0.630	0.330	0.114
	St29	30.12	32.35	15.0	0.290	0.570	0.470	0.026
	St30	30.29	32.36	21.0	0.220	0.420	0.170	0.038
	St31	30.18	32.33	24.0	0.250	0.230	0.130	0.036
	St32	30.20	32.37	20.5	0.210	0.300	0.170	0.024
	St33	29.65	32.40	21.0	0.280	0.370	0.270	0.021
	St34	29.49	32.85	16.0	0.260	0.430	0.170	0.024
	St42	30.07	32.62	13.5	0.480	0.670	0.430	0.033
	St43	29.99	32.74	16.5	0.480	0.480	0.370	0.029
	St44	29.86	32.80	20.0	0.380	0.300	0.180	0.017
	St45	29.44	32.81	15.0	0.390	0.430	0.300	0.060
	St46	28.95	33.19	15.5	0.350	0.430	0.220	0.075
	St47	29.09	33.19	14.0	0.410	0.370	0.200	0.036
	St48	28.99	33.17	20.0	0.430	0.270	0.130	0.036
	St49	30.25	32.39	19.5	0.290	0.530	0.270	0.026
	St50	30.25	32.41	11.5	0.580	0.780	0.450	0.029
	St51	30.58	32.27	10.5	0.510	0.770	0.520	0.058
	St52	29.88	30.15	3.0	1.820	4.420	3.270	0.171
August	St1	29.92	32.21	12.0	0.660	0.850	0.580	0.073
	St2	30.41	31.34	16.0	0.340	0.870	0.200	0.078
	St3	30.25	31.46	18.0	0.360	0.230	0.100	0.029
	St4	30.01	32.23	20.0	0.340	0.250	0.130	0.032
	St5	30.26	32.48	21.0	0.290	0.230	0.130	0.014
	St6	30.19	32.31	21.0	0.300	0.200	0.100	0.024
	St7	31.53	32.73	29.0	0.250	0.220	0.070	0.034
	St14	31.24	32.80	11.5	0.640	0.630	0.380	0.035
	St15	30.49	32.54	13.5	0.570	0.380	0.250	0.071
	St16	30.14	32.17	15.5	0.470	0.300	0.200	0.033
	St17	30.14	31.71	22.5	0.420	0.270	0.200	0.017
	St18	30.07	32.18	22.0	0.380	0.300	0.220	0.017
	St19	30.07	32.31	22.5	0.390	0.250	0.170	0.027
	St20	30.07	32.13	23.5	0.330	0.280	0.180	0.031
	St30	29.79	31.81	15.5	0.440	0.350	0.180	0.030
	St31	29.79	31.84	15.5	0.450	0.220	0.120	0.030
	St32	29.61	32.14	16.0	0.410	0.400	0.230	0.031
	St33	28.48	32.73	15.0	0.350	0.300	0.180	0.047
	St34	28.72	32.66	16.0	0.390	0.270	0.170	0.034
	St42	30.57	31.56	8.5	0.650	0.680	0.500	0.092
	St43	30.11	32.14	12.0	0.520	0.450	0.130	0.048
	St44	29.83	32.44	12.5	0.530	0.370	0.100	0.036
	St45	29.13	32.59	15.5	0.440	0.230	0.100	0.044
	St46	28.99	32.34	20.0	0.450	0.300	0.130	0.031
	St47	28.97	32.38	18.5	0.350	0.250	0.130	0.025
	St48	28.99	32.84	27.5	0.490	0.320	0.080	0.020
	St49	30.29	31.77	6.5	0.850	1.570	0.800	0.043
	St50	30.34	30.12	4.5	1.280	3.000	2.050	0.122
October	St1	29.86	31.58	13.0	0.830	0.750	0.530	0.093
	St2	29.88	32.06	14.0	1.040	0.780	0.510	0.051
	St3	29.80	31.87	12.0	0.900	0.960	0.630	0.046
	St4	29.78	32.05	15.0	0.560	0.940	0.510	0.053
	St5	29.64	32.39	22.0	0.380	0.330	0.200	0.027
	St6	29.86	32.28	20.5	0.370	0.640	0.400	0.020
	St7	30.03	32.22	20.0	0.340	0.640	0.360	0.047
	St14	30.02	32.25	11.5	1.020	0.810	0.570	0.049
	St15	29.89	32.48	14.5	0.500	0.900	0.660	0.054
	St16	30.06	32.56	11.5	0.480	0.840	0.670	0.047
	St17	29.97	32.50	13.5	0.760	0.840	0.580	0.052
	St18	29.80	32.65	15.0	0.520	0.680	0.470	0.057
	St19	29.59	32.81	17.0	0.410	0.670	0.390	0.072
	St20	29.86	31.81	15.0	0.390	0.460	0.220	0.069
	St28	29.92	30.64	8.5	1.330	1.530	1.140	0.102
	St29	30.05	31.21	8.5	1.230	1.320	0.950	0.105
	St30	29.82	32.44	10.0	0.650	1.040	0.800	0.070
	St31	29.79	32.59	16.5	0.520	0.600	0.140	0.033
	St32	29.71	32.70	26.0	0.360	0.420	0.210	0.032
	St33	29.87	32.73	28.0	0.270	0.350	0.130	0.046
	St34	29.71	32.81	30.5	0.190	0.360	0.130	0.019
	St42	29.62	31.84	12.5	1.430	0.830	0.530	0.096
	St43	29.75	32.16	15.0	1.020	0.800	0.520	0.078
	St44	29.67	32.63	22.0	0.590	0.590	0.280	0.026
	St45	29.67	32.72	24.0	0.360	0.530	0.140	0.032
	St46	29.85	32.67	26.5	0.300	0.330	0.150	0.020
	St47	29.91	32.66	30.0	0.150	0.280	0.180	0.034
	St48	29.74	32.72	31.5	0.110	0.280	0.060	0.017
	St49	29.54	32.06	10.0	0.770	1.050	0.900	0.072
	St50	29.49	31.78	10.5	0.650	0.980	0.760	0.079
	St51	29.63	31.22	8.5	0.980	1.000	0.790	0.083
	St52	29.53	30.52	3.5	2.340	2.830	2.300	0.088
November	St14	29.45	31.11	3.0	1.280	2.320	1.710	0.093
	St15	29.05	29.11	4.0	1.570	1.850	1.330	0.191
	St16	29.13	29.90	6.0	1.140	0.880	0.500	0.147
	St17	29.20	30.09	7.0	1.070	0.900	0.500	0.131
	St18	29.11	30.49	8.0	1.060	0.700	0.610	0.124
	St19	29.22	31.41	13.5	0.600	0.620	0.290	0.044
	St20	29.60	32.61	15.0	0.560	0.530	0.210	0.027
	St28	29.04	25.62	3.0	3.100	5.130	3.000	0.291
	St29	28.65	25.95	4.0	1.900	4.070	3.030	0.264
	St30	28.91	30.35	6.0	1.640	0.600	0.280	0.132
	St31	29.37	30.99	7.0	1.050	0.700	0.300	0.071
	St32	30.13	31.95	17.0	0.620	0.430	0.160	0.021
	St33	30.39	32.49	15.0	0.460	0.300	0.210	0.015
	St34	30.00	32.78	10.0	0.650	0.620	0.310	0.057
	St42	28.98	28.14	6.0	1.450	1.600	0.650	0.190
	St43	28.81	28.25	11.0	1.040	0.850	0.460	0.189
	St44	29.20	30.76	16.0	0.970	0.720	0.250	0.071
	St45	29.32	31.53	16.0	0.860	0.420	0.160	0.055
	St46	29.65	32.09	20.0	0.520	0.420	0.150	0.041
	St47	29.45	32.47	20.0	0.480	0.470	0.190	0.043
	St48	29.60	32.65	16.0	0.560	0.530	0.220	0.029
	St49	29.39	28.04	6.0	1.580	1.300	0.830	0.231
	St50	29.34	25.94	3.0	1.580	6.200	4.810	0.268
	St51	29.17	25.23	3.0	2.690	9.000	6.450	0.321
	St52	28.19	22.29	1.0	7.740	25.800	18.800	0.408

The interrelationships among certain biophysical parameters are presented in Fig. 2. These relationships indicate a strong non-linear correlation among optically active constituents (Chl-a, SPM, MSS and CDOM); and between SDD and optically active constituents.

## 2 Experimental design, materials, and methods

### 2.1 Experimental design

**Fig. 3:**
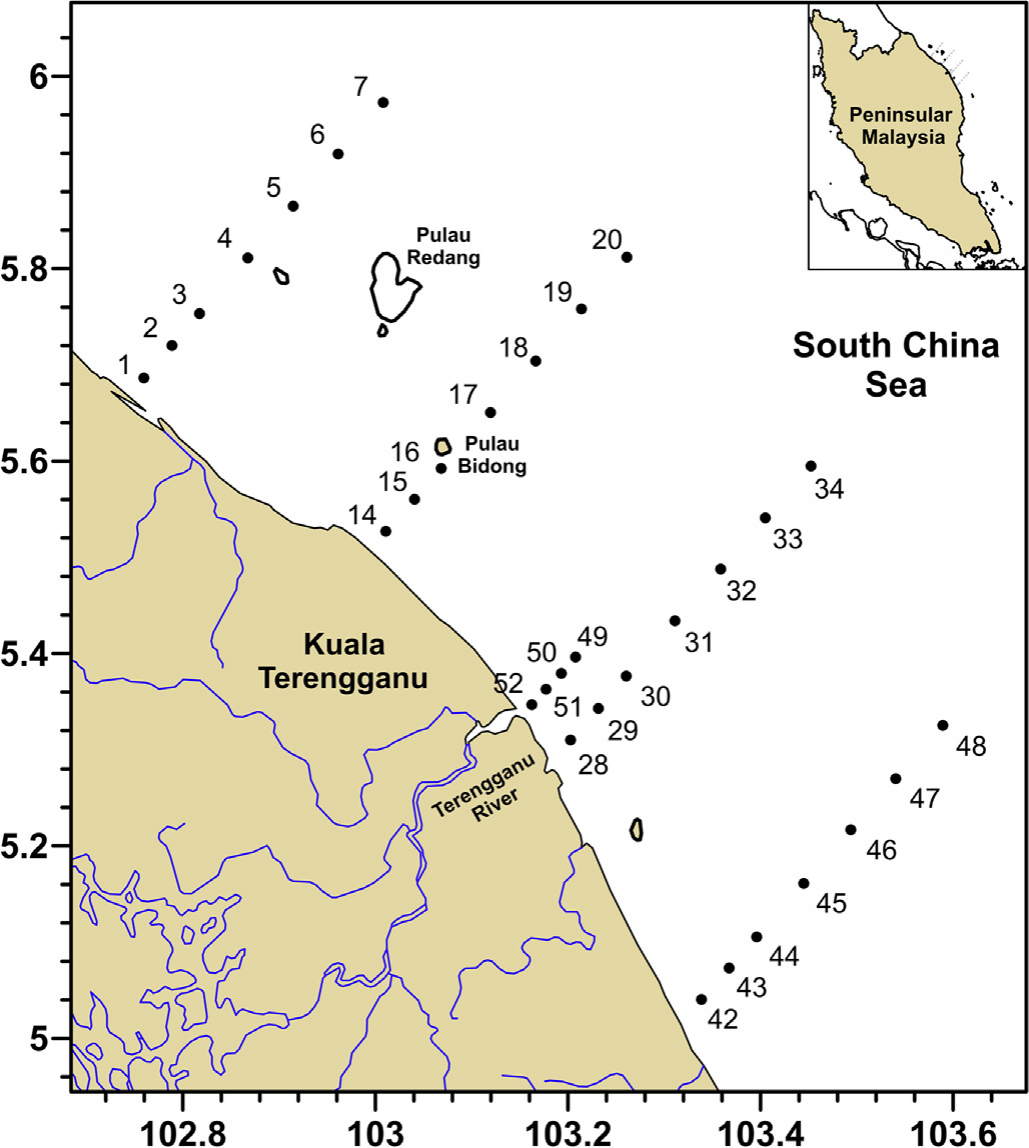
Location map of the sampling stations in the east coast of Peninsular Malaysia.

The stations were sampled between May 2009 and November 2009 under various environmental conditions during different monsoon seasons. The sampling dates (Table 3) coincide with 3 major monsoon seasons, the southwest monsoon (June to August), northeast monsoon (November) and inter-monsoon (May and October). Although it is difficult to determine the actual timing of each monsoon onset, the monsoon intra-seasonal oscillation is relatively repeatable with each monsoon onset can vary by two to three weeks from year to year [1,2]. The data were collected along inshore-offshore transects that extend from 5.04° N to 5.97° N latitudes and 102.76° E and 103.59° E longitudes (Table 1 and Fig. 3). The distance between the sampling stations for each transect varies from 5 km at the coastal stations (the first 3 stations) to 9 km at the seaward stations (Fig. 3). All stations were sampled during each cruise except for stations 1–7 in May and October due to weather conditions. For all measurements, water samples were collected from approximately 0.2–0.3 m depth using a submersible water pump into 10 l dark bottles. The entire sampling was done in the daytime only (9.30 a.m.–4.00 p.m.).

**Table 3:** Dates of in situ measurements.

Start date	End date	Description
May 13, 2009	May 18, 2009	Spring inter-monsoon; Relatively clear water; cloudy
June 14, 2009	June 18, 2009	Southwest monsoon; Relatively clear water; clear sky
July 6, 2009	July 9, 2009	Southwest monsoon; Relatively clear water; cloudy
Aug 11, 2009	Aug 14, 2009	Southwest monsoon; Relatively clear water; cloudy
Oct 21, 2009	Oct 24, 2009	Fall inter-monsoon; Relatively clear water; cloudy
Nov 10, 2009	Nov 12, 2009	Northeast monsoon; A week after heavy rain event, cloudy

### 2.3 Oceanographic parameters

Basic oceanographic parameters of temperature and salinity were measured at each sampling station using two different types of instrument; a SBE 911 CTD profiler and a Yellow Springs Instruments (YSI) 6600 Multi Parameter V2 Sonde. In situ Secchi disk depth (SDD) was determined at each station using a conventional black and white Secchi disk with a diameter of 50 cm.

### 2.4 Chlorophyll-a

At each sampling station, only one replication of water sample was collected for the chlorophyll-a measurement. Chlorophyll-a concentration was determined spectrophotometrically using a Cary-100 double beam Spectrophotometer (Agilent Technologies). A known volume of water (1–5 L depending on particle load) was filtered under low vacuum pressure (300–400 mmHg) onto 47-mm Whatman glass-fibre filters (GF/F) with pore size 0.7 μm. Samples were subsequently kept on ice after collection and stored in the dark until analysis in the laboratory. The particulate matter retained on the filters was extracted in 10 ml volume of 90% acetone and refrigerated between 8 and 24 hours. The trichromatic equations [3] were used to calculate concentrations of chlorophyll.

### 2.5 Suspended particulate matter and mineral suspended solid

Three replicates of water samples were collected from surface water at each station. Filters were washed with 250 ml of distilled water after filtration to remove any trace of salt and were immediately stored cooled until analyzed. Suspended particulate matter (SPM) was measured gravimetrically on pre-weighed and pre-combusted 0.7 μm GF/F filters (450 °C for 4 hours). Filters were oven-dried at 75 °C for 24 hours [4], cooled to room temperature and reweighed on the same balance (0.1 mg precision) to obtain SPM. The filters were then re-combusted at 450 °C for 4 hours and reweighed again to obtain mineral suspended solid (MSS).

### 2.6 Chromophoric dissolved organic matter (CDOM)

Samples for CDOM were collected by filtering 150–200 ml of water through 0.2 μm Whatman Nucleopore polycarbonate filters into pre-acid washed and pre-combusted amber glass bottles [4]. For this parameter, only one sample was collected for each station. The filtrates were stored frozen for analysis in the laboratory and were analyzed within 1 week of collection. CDOM absorption (a_g_) was measured using a Cary-100 dual beam Spectrophotometer using a 10 cm cylinder cuvette and corrected with a Milli-Q blank. The measured absorbance data were normalized to zero at wavelengths between 750 and 800 nm to remove temperature related measurement artifacts [5]. The absorption coefficient at 443 nm was selected as a reference wavelength to represent the CDOM concentration as calculated from Eq. (1).

(1) $${\text{a}}_{\text{g}}\left(443\right)=\;2.303\left({\text{A}}_{443}-{\text{A}}_{750}\right)/0.1\;$$

where A_443_ and A_750_ are the absorbances measured at 443 and 750–800 nm, respectively. The constant of 2.303 is a conversion factor to convert natural log to the base 10 and 0.1 is the cell path length of the cylindrical cuvette in meters.

## References

[1] Lau K., Yang S. (1997). Climatology and interannual variability of the southeast Asian summer monsoon. Adv. Atmos. Sci..

[2] Hoyos C.D., Webster P.J. (2007). The role of intraseasonal variability in the nature of Asian monsoon precipitation. J. Clim..

[3] Jeffrey S.W., Humphrey G.F. (1975). New spectrophotometric equations for determining chlorophylls a, b, c in higher plants, algae and natural phytoplankton. Biochem. Physiol. Pflanz. (BPP).

[4] Mueller J.L., Mueller J.L., Fargion G.S., McClain C.R. (2003). And others, Ocean optics protocols for satellite ocean color sensor validation.

[5] Pegau W.S., Zaneveld J.R.V. (1993). Temperature-dependent absorption of water in the red and near-infrared portions of the spectrum. Limnol. Oceanogr..

